# Passive Experimental Autoimmune Encephalomyelitis in C57BL/6 with MOG: Evidence of Involvement of B Cells

**DOI:** 10.1371/journal.pone.0052361

**Published:** 2012-12-26

**Authors:** Francesco Mannara, Tony Valente, Josep Saura, Francesc Graus, Albert Saiz, Beatriz Moreno

**Affiliations:** 1 Center for Neuroimmunology, Service of Neurology, Hospital Clinic and Institut d'Investigació Biomèdica August Pi I Sunyer (IDIBAPS), Barcelona, Spain; 2 Biochemistry and Molecular Biology Unit, School of Medicine, University of Barcelona, Barcelona, Spain; Institute Biomedical Research August Pi Sunyer (IDIBAPS) - Hospital Clinic of Barcelona, Spain

## Abstract

Experimental autoimmune encephalomyelitis (EAE) is the most relevant animal model to study demyelinating diseases such as multiple sclerosis. EAE can be induced by active (active EAE) or passive (at-EAE) transfer of activated T cells in several species and strains of rodents. However, histological features of at-EAE model in C57BL/6 are poorly described. The aim of this study was to characterize the neuroinflammatory and neurodegenerative responses of at-EAE in C57BL/6 mice by histological techniques and compare them with that observed in the active EAE model. To develop the at-EAE, splenocytes from active EAE female mice were harvested and cultured in presence of MOG_35–55_ and IL-12, and then injected intraperitoneally in recipient female C57BL6/J mice. In both models, the development of EAE was similar except for starting before the onset of symptoms and presenting a higher EAE cumulative score in the at-EAE model. Spinal cord histological examination revealed an increased glial activation as well as more extensive demyelinating areas in the at-EAE than in the active EAE model. Although inflammatory infiltrates composed by macrophages and T lymphocytes were found in the spinal cord and brain of both models, B lymphocytes were significantly increased in the at-EAE model. The co-localization of these B cells with IgG and their predominant distribution in areas of demyelination would suggest that IgG-secreting B cells are involved in the neurodegenerative processes associated with at-EAE.

## Introduction

Experimental autoimmune encephalomyelitis (EAE) is a CD4^+^ T cell-mediated inflammatory demyelinating disease of the central nervous system (CNS). Although no animal model recapitulates all the underlying mechanisms of a complex human disease, the EAE is the most commonly used experimental model for multiple sclerosis (MS). There is a great heterogeneity in the susceptibility and method of induction, also in the response to immunological or neuropharmacological interventions [1]–[3]. This makes EAE a very versatile system to use in translational neuroimmunopharmacology, but the model needs to be tailored to the scientific question being asked. EAE can be induced in many susceptible species using several routes. Initially, animals were actively immunized with self-antigen in the form of myelin components or CNS homogenates emulsified in adjuvant [4]–[10]. Later on, EAE was passively induced by adoptive transfer (at-EAE) of encephalitogenic T cells with specificity to self–antigens [6], [8], [11]–[13]. More recently, spontaneous EAE models have been also developed using transgenic [14], [15]. Active EAE is the easiest inducible model giving fast results in screening the effects of drugs on autoimmune inflammation. Years ago, Lewis rats were the most popular animals used for EAE due mainly to their 100% responsiveness after immunization with myelin basic protein (MBP) [9]. Nowadays, C57BL/6 mice have become more popular mainly for studies involving transgenic mice [14]. at-EAE is a very useful model to answer questions related with the effector phase of the disease. The encephalitogenic T cells can also be manipulated *in vitro* to study the role of specific cytokines and other biological agents before transfer to recipients. These cells can be labeled to follow their localization, survival or interactions with other cell types in the recipient. Moreover, the at-EAE model is of particular interest to study the role of a variety of inflammatory molecules in different aspects of disease development and regulation through the use of gene-targeted donor or recipient animal strains [1], [2].

Due to the lack of extensive histological studies in C57BL/6 comparing active and passive transfer EAE models when mice are immunized with MOG_35–55_ is important to characterize by histological techniques the neuroinflammatory and neurodegenerative responses taking into account the role not only of T cells but also B cells which appear to have a more significative role in the passive model.

## Materials and Methods

### Animals and EAE Model

C57BL/6J mice were purchased from Harlan Iberica (Spain) and maintained under regulated light and temperature conditions at the animal facilities of the Faculty of Pharmacy, University of Barcelona. Animal experiments were done in accordance with the Guidelines of the European Union Council (86/609/EU) and Spanish Government (BOE 67/8509-12), and approved by the Ethics and Scientific Committees from the University of Barcelona and registered at the “Departament d’Agricultura, Ramaderia i Pesca de la Generalitat de Catalunya”. Sixty-eight female mice 6–8 weeks old were immunized with an encephalitogenic cocktail containing MOG_35–55_ (150 µg/mouse) and H37R Mycobacterium tuberculosis (1 mg/mouse) in complete Freund’s adjuvant (CFA). Female mice were injected intraperitoneally (i.p.) with pertussis toxin from Bordetella pertussis (500 ng/mouse), 1 h and 48 hours after immunization. Mice were weight each day after immunization and EAE symptoms (mobility loss and hind limb paralysis) were evaluated from day five after immunization in according with this score scale: 0 = no symptoms; 1 = total loss of tail tonicity; 2 = difficulty in righting; 3 = unsteady gait and mild paralysis; 4 = total hind-limb paralysis and incontinence; 5 = total paralysis; 6 = moribund or death. During EAE score evaluation none animal was sacrificed, since the score did not reach 5. Mice with a score of 4 had hind leg paralysis and incontinence on day 17 after immunization. However, on the day of sacrifice (day 19) improvement was observed in mice and the mean score was less than 3.5. Wet food and easier access to fresh water was supplied when the animals had paralysis of one or two of the hind legs. Moreover, if a mouse had less than 16 grams of weight, it was administered daily with 200 µl of saline glycosylated solution. On day 19 after immunization, mice were sacrificed by an overdose of anesthesia (ketamine and xylazine, 100 and 20 mg/kg respectively).

### Adoptive Transfer of Experimental Autoimmune Encephalomyelitis (at-EAE Model)

To develop the at-EAE, spleens from active female EAE mice (mean score = 2.8±0.2) were extracted 12 days after immunization. Spleens were dissected and passed through a 100 µm filter (Falcon^TM^) to obtain a single cell suspension. Cells were incubated with a red blood cells lysis buffer (Ammonium chloride NH_4_Cl 155 mM, potassium bicarbonate KHCO_3_ 10 mM, EDTA pH 8.0 0,01 mM) for 5′ at 4°C. After one centrifugation (500 rcf 5′ 4°C), cells were cultured in presence of MOG_35–55_ (50 µg/ml) and IL-12 (25 ng/ml) in a concentration of 5·10^6^ cells/mL. After 72 hours, 20·10^6^ splenocytes/mouse were injected i.p. in 33 recipient female C57BL/6J mice with 7 weeks old (18–20 g) to induce at-EAE. The experiment was repeated 3 times. In parallel, six others female C57BL/6J mice were immunized to develop an active EAE used as positive control, and 3 CFA and 3 untreated mice as negative control. On day 19 after cells injection, animals were anesthetized and perfused with 4% of PFA (paraformaldehyde). The brains and lumbar spinal cord were carefully removed, post-fixed in PFA for 48 h, paraffin processed, cut in 5 µm sections and neurohistological techniques were performed. Three mice were sacrificed on day 46 after treatment to evaluate the long-term evolution of at-EAE symptoms.

### Histology

Four sequential sections from each case, controls and immunized mice, were deparaffinised, hydrated, and washed in PBS and then stained for each histological procedure. For hematoxylin and eosin staining, we used the standard protocol for Harris hematoxylin. For luxol fast blue technique, slides were stained with luxol fast blue solution at 56°C overnight, washed with 95% ethanol and distilled water. After that, slides were differentiated in lithium carbonate solution (0.05%) and 70% ethanol for 30 seconds in each solution. After several washes in distilled water, slides were ethanol dehydrated and coverslipped with DPX and analyzed under bright light microscope. Alternatively, some sections were counterstained with cresyl violet solution. For the FluoroJade histofluorescent staining we used the protocol from Millipore. Briefly, the slides were washed in distilled water and incubated in a 0.06% potassium permanganate solution for 15 min. After that, they were incubated in a 0.001% FluoroJade staining solution for 30 min. Following the fluorescent staining, the slides were washed in distilled water and dried at 50°C. Then they were immersed in xylene and coverslipped with DPX, and microscopically analyzed under fluorescent/FITC filter.

### Immunohistochemistry

Sections were deparaffinised, hydrated, and washed in PBS containing 0.1% triton (PBS-T). Antigen retrieval was performed incubated the sections in citrate buffer (pH = 6.0) at 95°C during 30 minutes. Then, sections were rinsed in citrate buffer and PBS at room temperature. After that, the sections were treated with 2% H_2_O_2_ in methanol during 10 minutes, rinsed in PBS-T, blocked with 10% of fetal bovine serum and incubated overnight with one of the following primary antibodies: anti-GFAP (1∶300, Dako), anti-CD3 (1∶100, Abcam), anti-CD-45R (1∶100, BD Pharrmingen), anti-CD-138 (1∶100, Abcam), anti-MBP (1∶300, Millipore), anti-MOSP (1∶500, Millipore), anti-NeuN (1∶100, Sigma-Aldrich), anti-MAP2 (1∶500, Sigma-Aldrich), anti-caspase-3-cleaved (1∶100, Millipore), anti-IgG (1∶100, Thermo Scientific). Sections were then incubated with biotinylated goat anti-rabbit or mouse antibody (1∶500, Sigma) and then with ExtrAvidin-HRP (1∶500, Sigma-Aldrich). For microglia and macrophages staining we used biotinylated tomato isolectin (1∶200, Sigma-Aldrich) and then the sections were incubated with ExtrAvidin-HRP (1∶500, Sigma-Aldrich). Finally, the sections were developed with 0.05% diaminobenzidine-0.01% H_2_O_2_ (brown colour stain), washed in PBS-T and mounted in DPX medium. Alternatively, some sections were counterstained with methyl green.

### Immunopositive Cell Counting and Semiquantitative Histological Analysis

Two observers, blinded to the experimental methodology, analyzed all sections in a light microscope Eclipse 901 (Nikon) and images were captured with a digital camera (Nikon). All immunopositive cells in the gray or white matter of lumbar spinal cord were counted in four randomly chosen sections for each mouse of each group: control (n = 4), active EAE (n = 7), and at-EAE (n = 10). Semiquantitative histological evaluation for glial markers (GFAP, isolectin and Iba-1; 40× magnified microscope field), cell infiltration markers (CD-45R and CD3; 40× magnified microscope field), demyelination markers (MOSP and MBP; 10× magnified microscope field), neuronal markers (NeuN and MAP-2; 10× magnified microscope field) and neurodegeneration marker (FluoroJade; 20× magnified microscope field) was conducted and scored blindly using the following scale: 0 = absent of label or no positive cells, 1 = very weak label or less than 5 positive-cells/microscope field, 2 = weak label or 5 to 10 positive cells/microscope field, 3 = moderate label or 10 to 15 positive cells/microscope field, 4 = strong label or 15 to 20 positive cells/microscope field, 5 = very strong label or more than 20 positive cells /microscope field. For NeuN and CD-45R, the histological score increased to 6 due to high cell density was more than 50 cells per microscope field.

### Double Immunofluorescence

For double immunohistofluorescence, the sections were deparaffinised, hydrated, washed in PBS and incubated in citrate buffer (pH = 6.0) at 95°C during 30 minutes. After, several washes in PBS, the sections were blocked with 10% of FBS and incubated overnight at 4°C with two of following primary antibodies: anti-CD3 (1∶100, Abcam), anti-CD-45R (1∶100, BD Pharrmingen), anti-CD-138 (1∶100, Abcam) or anti-IgG (1∶500, Vector). After rinsing in PBS-T, sections were incubated 1 hour at room temperature with goat anti-rabbit ALEXA 546 (1∶500) and/or goat anti-rat and/or anti-mouse ALEXA 488 (1∶500) secondary antibodies and DAPI. For IgG immunolabelling, the sections were incubated first with goat anti-mouse biotinylated and then with ExtrAvidin-ALEXA 488 or 546. Finally, after washed in PBS-T the sections were mounted in mowiol medium.

### Data Presentation and Statistical Analysis

Histological techniques were repeated at least 3 times for each independent experiment (n = 3). All statistical analyses were performed using Kruskal-Wallis nonparametric test followed by Dunn’s post analysis. The data were presented as mean values ± SEM. Values of p<0.05 were considered statistically significant. All statistical analyses were performed by GraphPad Prism software (GraphPad Software, Inc., La Jolla, USA).

## Results

### Clinical Development of the at-EAE

**Figure 1:**
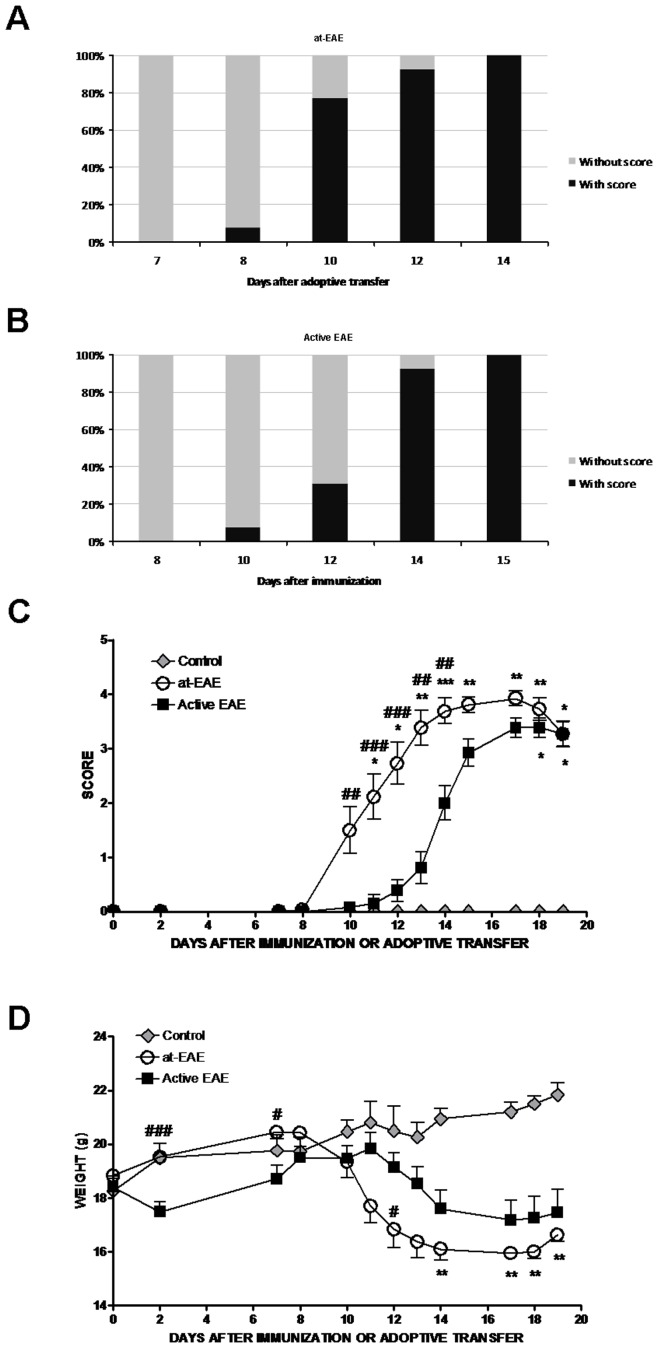
EAE induction in C57/BL6 mice by adoptive transfer of encephalitogenic cells (at-EAE) or active immunization (active EAE). Incidence in at-EAE mice (A) and active EAE mice (B) between day 7 and 15 after immunization or adoptive transfer. (A) at-EAE symptoms appeared on day 8 after immunization and the 100% of incidence is reached on day 14. (B) Active EAE symptoms appeared on day 10 after cells injection and the 100% of incidence is reached on day 15. (C) Average score of at-EAE and active EAE from the day of immunization or adoptive transfer to day 19. Increasing the score in at-EAE mice and active EAE is significant with respect to control mice (at-EAE: day 11, *, p<0.05; day 12, *, p<0.05; day 13, **, p<0.01; day 14, ***, p<0.001; day 15, **, p<0.01; day 17, **, p<0.01; day 18, **, p<0.01; 19, *, p<0.05; active EAE: day 18, *, p<0.05; day 19, *, p<0.05). After immunization or adoptive transfer score is significantly higher in at-EAE than in active EAE (day 10, ##, p<0.01; day 11, ###, p<0.001; day 12, ###, p<0.001; day 13 ##, p<0.01; day 14, ##, p<0.01). (D) Weight variations during EAE development. Weight loss in at-EAE mice is significant with respect to control mice between days 14 and 19 (day 14, **, p<0.01; day 17, **, p<0.01; day 18, **, p<0.01; and day 19, **, p<0.01). Significant weight differences are observed between at-EAE and active EAE on day 2 (###, p<0.001), day 7 (#, p<0.05) and day 12 (#, p<0.05). Data are representative of three separate experiments. Each group represents the mean of 6 mice for control, 13 mice for both at-EAE and active EAE, with SEM error bars. All statistical analyses were performed using Kruskal-Wallis nonparametric test followed by Dunn’s post analysis. Values of p < 0.05 were considered statistically significant.

Mice injected with MOG_35–55_ activated T cells developed the first neurological symptoms at day 8 reaching 100% of incidence on day 14 (Figure 1A, C), while in the active EAE neurological symptoms starting two days later and reaching 100% of incidence on day 15 (Figure 1B, C). At 100% of incidence, all the animals showed progressive weight loss, limp tail, and mild-to-moderate paraparesis. The loss of weight in the at-EAE animals occurred at the same time of the first clinical symptoms, while in the active EAE the weight loss follows two days after the appearance of the symptoms (Figure 1C, D).

The mean maximum score reached during the time course of the experiment was 4.08±0.08 in at-EAE animals and 3.69±0.18 in active EAE (Table 1). However, this difference in the mean maximum score was not statistically significant. By contrast, the mean scores at the end of the experiment were significantly higher (p<0.01) in at-EAE than in active EAE (3.29±0.11 and 2.75±0.15, respectively, Table 1). Therefore, the cumulative EAE score was significantly higher (p<0.001) in at-EAE than in active EAE (28.19±1.69 and 16.38±1.34, respectively, Table 1).

**Table 1:** at-EAE versus active EAE models.

EAE model	n	Incidence	EAE day onset	EAE score	EAE maximum score	EAE cumulative score
atEAE	13	EAE day 14 (100 %)	10.4 ± 0.4	3.3 ± 0.1	4.1 ± 0.1	28.2 ± 1.7
Active EAE	13	EAE day 15 (100 %)	13.0 ± 0.4	2.8 ± 0.2	3.7 ± 0.2	16.4 ± 1.3
			***	**	NS	***

** , p<0.01; ***, p<0.001; NS, not statistically significant; Kruskal-Wallis test (Dunn’s post analysis).

### Glial Activation

**Figure 2:**
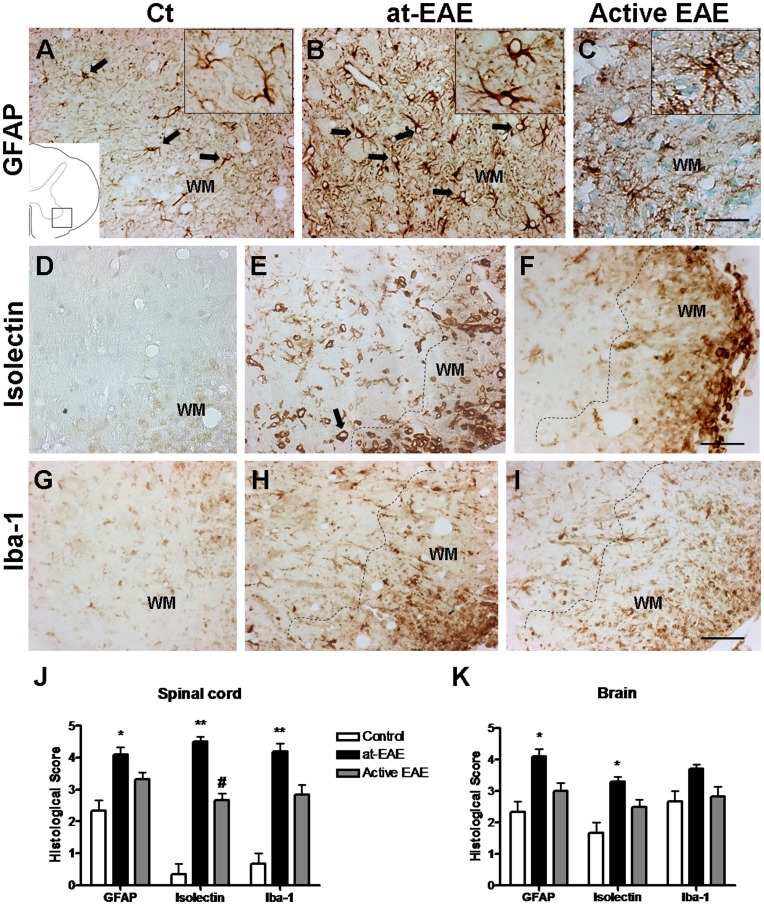
Glial activation in lumbar spinal cord. Astroglial GFAP immunostaining is observed in lumbar spinal cord of at-EAE mice (B) and active EAE mice (C) when compared with control (A). Isolectin labeling in the lumbar spinal cord show increase in at-EAE mice (E) compared with active EAE (F) and control mice (D). Iba-1 immunostaing (microglial marker) is up-expressed in the lumbar spinal cord of at-EAE mice (H) compared with active EAE (I) and control mice (G). Semiquantitative histological analysis of glial markers in lumbar spinal cord (J) and in ventricular regions of brain (K). A significant increase of glial-positive cells is observed in at-EAE versus control both in lumbar spinal cord (GFAP: *, p<0.05; Isolectin and Iba-1: **, p<0.01) and brain (GFAP and Isolectin: *, p<0.05). The increased of Isolectin-positive cells is significantly higher in at-EAE than in active EAE (#, p<0.05). Data are representative of three separate experiments. Each group represents the mean of 4 mice for control, 10 mice for at-EAE and 7 mice for active EAE, with SEM error bars. All statistical analyses were performed using Kruskal-Wallis nonparametric test followed by Dunn’s post analysis. Values of p < 0.05 were considered statistically significant. Magnification bar 150 µm.

Histological examination of the CNS tissue from the at-EAE showed an increase in the immunolabeling of glial markers, both for astrocytes and microglia/macrophages in comparison with control animals (Figure 2). GFAP staining showed hypertrophic astrocytes in spinal cord of at-EAE and active EAE animals. No significant differences in GFAP staining were found between at-EAE and active EAE (Figure 2A–C, J). Moreover, the presence of microglia/macrophages with amoeboid morphology stained by isolectin (Figure 2D–F) and Iba-1 (Figure 2G–I) evidenced an undergoing inflammatory process in both models. However, isolectin staining was significantly increased in at-EAE compared with the active model (Figure 2 D–J), although the glial activation was more discrete in the brain that in the spinal cord (Figure 2K).

### Demyelination and Neuronal Damage

**Figure 3:**
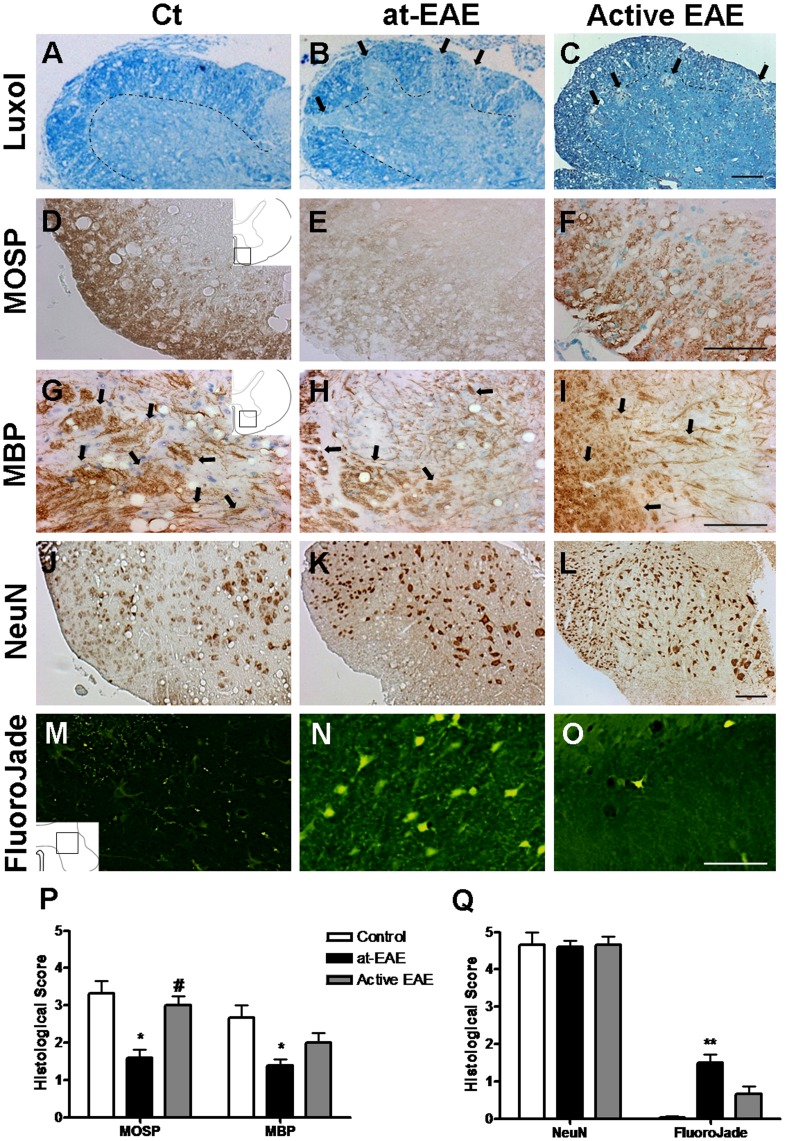
Demyelination and neurodegeneration markers in the lumbar spinal cord. The demyelination is clearly demonstrated by a loss of signal in the luxol fast blue staining (A–C) and MOSP (D–F) and MBP (G–I) immunostaining of at-EAE (B, E and H, respectively) and active EAE mice (C, F and I, respectively) when compared with control mice (A, D and G, respectively). NeuN immunostaining in control (J), at-EAE mice (K) and active EAE (L). None FluoroJade staining is observed in control mice (M), while some FluoroJade positive cells are observed in at-EAE mice (N) and few positive cells are observed in active EAE mice (O). Semiquantitative histological analysis of demyelination (P) and neurodegeneration (Q) markers in lumbar spinal cord. A significant decrease of immunostaining is observed in at-EAE versus control in lumbar spinal cord (MOSP and MBP: *, p<0.05). The decrease of MOSP immunostaining in at-EAE mice is significant with respect to the active EAE mice (#, p<0.05). No changes are detected in the NeuN-positive cell number meanwhile an increase in the number of degenerating neurons (FluoroJade) is observed in at-EAE mice with respect to control mice (**, p<0.01). Data are representative of three separate experiments. Each group represents the mean of 4 mice for control, 10 mice for at-EAE and 7 mice for active EAE, with SEM error bars. All statistical analyses were performed using Kruskal-Wallis nonparametric test followed by Dunn’s post analysis. Values of p<0.05 were considered statistically significant. Magnification bar 200 µm.

Luxol fast blue staining showed demyelinated areas in the lumbar spinal cord of at-EAE and active EAE mice (Figure 3A–C). Immunostaining for MOSP (Figure 3D–F) and MBP (Figure 3G–I), markers for oligodendrocytes and myelin respectively, showed a decrease in the intensity of the markers in both at-EAE and active EAE models in comparison with controls. Furthermore, MOSP quantification was significantly lower in the at-EAE in comparison with the active model or control mice indicating more extreme demyelination in the spinal cord of at-EAE (Figure 3P). In contrast, we did not observe demyelination in the brain (data not shown). In order to evaluate neuronal damage, we performed NeuN staining in the spinal cord of the animals. We found no neuronal loss in at-EAE mice although an up-regulation of NeuN protein was observed (Figure 3J–L). Then we performed caspase-3-cleaved immunohistochemistry and we did not detect apoptotic cells confirming the previous result (data not shown).

FluoroJade stain was also used in order to label degenerating neurons (Figure 3M–O). In the at-EAE model neuronal loss was not detectable by NeuN staining but when FluoroJade was used a large number of neurons was stained indicating that, even though neurons were not dead, they had begun a degenerative process (Figure 3Q). In the active EAE model a significant lower number of positive cells was observed in comparison with the at-EAE model (Figure 3N–O).

### Cell Infiltration in the CNS

**Figure 4:**
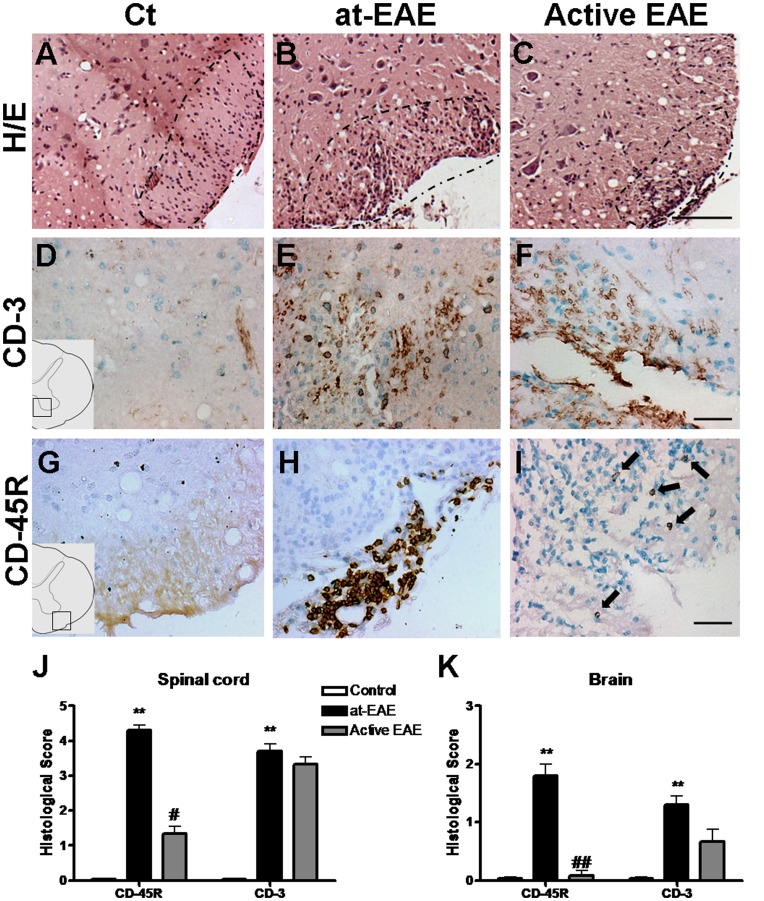
Lymphocyte infiltrates in lumbar spinal cord. H/E technique show absence of blood infiltrates in control mice (A) while many infiltrates are observed in at-EAE (B) and active EAE mice (C). No T cells (CD3 marker) immunostaining is observed in lumbar spinal cord of control mice (D). Many T cells are detected in the parenchyma of at-EAE mice and active EAE (E and F, respectively). No B-cells (CD-45R marker) immunostaining is observed in lumbar spinal cord of control mice (G). Many B cells are observed mainly in at-EAE mice (H) while few B-cells are detected in active EAE (I). Semiquantitative histological analysis of infiltration markers in lumbar spinal cord (J) and brain (K). A significant increase of CD-45R-positive cells is detected in at-EAE mice with respect to control mice both in lumbar spinal cord (**, p<0.01) and brain (**, p<0.01). The increase of CD-45R-positive cells was higher in at-EAE than in active EAE , both in lumbar spinal cord (#, p<0.05) and in brain (##, p<0.01). An increase in the number of CD3-positive cells has been observed in at-EAE in both CNS areas (**, p<0.01). Data are representative of three separate experiments. Each group represents the mean of 4 mice for control, 10 mice for at-EAE and 7 mice for active EAE, with SEM error bars. All statistical analyses were performed using Kruskal-Wallis nonparametric test followed by Dunn’s post analysis. Values of p < 0.05 were considered statistically significant. Magnification bar 150 µm.

In the lumbar spinal cord of at-EAE mice and active EAE, H/E staining showed inflammatory infiltrates mainly localized around the central canal and marginal zone (overlapping the demyelination areas) (Figure 4A–C). The localization and size of the infiltrates was similar in both models. However, the number of infiltrates was higher in at-EAE. To study the composition of the inflammatory infiltrates we immunostained for different cell markers. As shown previously (Figure 2), some macrophages from the periphery were present in the inflammatory infiltrates. CD3 (T lymphocytes) and CD-45R (B lymphocytes) positive cells were also present in the inflammatory infiltrates (Figure 4D–F and G–I, respectively). Semiquantitative histological analysis showed that in both EAE models the number of T cells (CD3 positive cells) in the infiltrates was similar but B cells (CD-45R positive cells) were significantly increased in the at-EAE in comparison with the active EAE model, mainly in demyelination areas (Figure 4J). Similar results were obtained in the brain of at-EAE mice; however, the total number of inflammatory infiltrates was lower than in the spinal cord (Figure 4J–K).

### Involvement of B Cells in the Pathogeneses of the at-EAE Model

**Figure 5:**
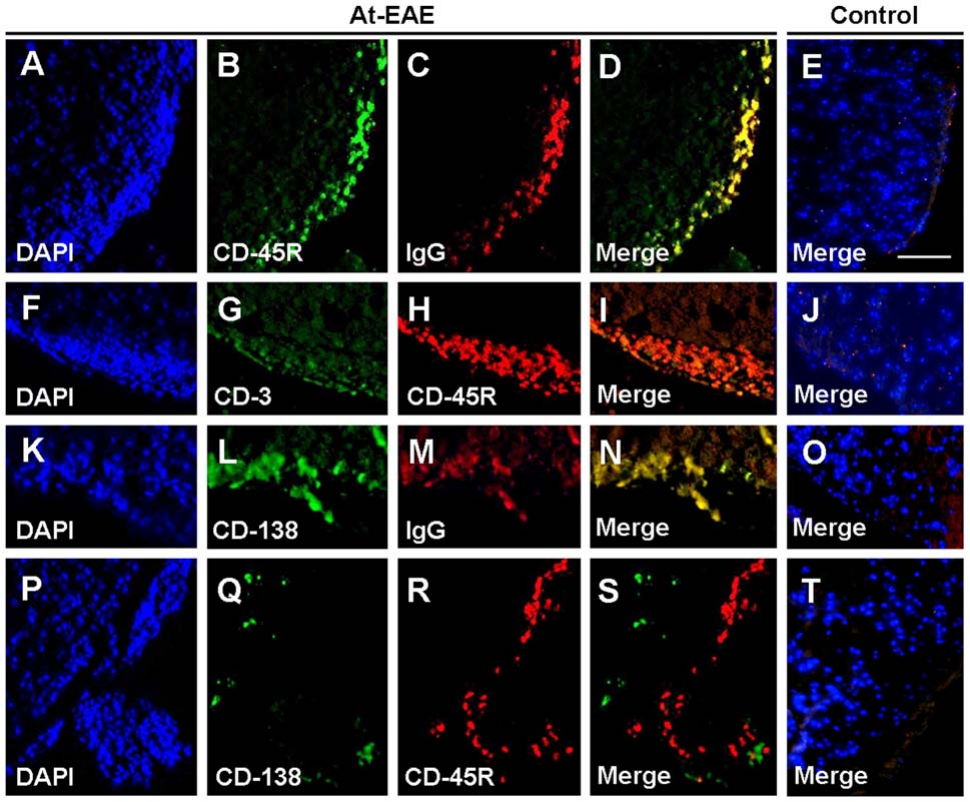
Double immunostaining for B cells in at-EAE and control spinal cord sections. DAPI (A), CD-45R (B), IgG (C), merge (D) at-EAE fluorostaining sections, and control merge (E) fluorostaining sections. DAPI (F), CD3 (G), CD-45R (H), merge (I) at-EAE fluorostaining sections, and control merge (J) fluorostaining sections. DAPI (K), CD-138 (L), IgG (M), merge (N) at-EAE fluorostaining sections, and control merge (O) fluorostaining sections. DAPI (P), CD-138 (Q), CD-45R (R), merge (S) at-EAE fluorostaining sections, and control merge (T) fluorostaining sections. Magnification bar 150 µm.

Although CD-45R is widely used as a marker for B cells, it has been reported that CD-45R can also be found in some subsets of activated T cells [16], [17]. For this reason, we studied whether these CD-45R-positive cells found in at-EAE were antibodies producing cells by immunocolocalization with mouse IgG. The results obtained showed that most of the CD-45R-positive cells co-localized with IgG (Figure 5A–D), and only a few cells co-localized with CD3 (Figure 5F–I), suggesting that most of them were IgG secreting cells. In addition, we used syndecan 1 (CD-138), another B cell marker which selectively stains plasma cells (antibodies producing cells), and we found that many of the CD-138-positive cells co-localized with mouse IgG (Figure 5K–N) but not with CD-45R-positive cells (Figure 5P–S), suggesting the existence of two distinct B cell subpopulations. In control mice, there were no cells stained by these markers (Figure 5 E, J, O, T).

## Discussion

In the current study we developed and characterized by histological techniques an at-EAE model in C57BL/6 mice using the MOG35-55 peptide and IL-12. This at-EAE was a very reproducible model with high incidence, in contrast with Cravens and colleagues conclusions arguing that this mouse strain is relatively resistant to this method of EAE induction [18]. The disease presented as a typical EAE, similar to that described after injection with MBP or PLP [19]. The development of EAE was similar in both transfer and active models except for the earlier onset of symptoms and the higher EAE cumulative score in the at-EAE. However, the most striking differences were the increased glial activation as well as more extensive areas of demyelination and neurodegenerative changes in the lumbar spinal cord of at-EAE. In addition, although the inflammatory infiltrates composed by macrophages and T lymphocytes were similar in both models, B lymphocytes were significantly increased in the at-EAE model. The co-localization of these B cells with IgG is a finding not reported previously and suggests the presence of antibody-secreting cells. Moreover, these IgGs-secreting B cells were restricted to degenerating areas in which we demonstrated to have a glial activation (GFAP, Isolectin and Iba-1 immunostaining), demyelination (MOSP and MBP immunostaining) and a neurodegeneration (FluoroJade staininig).

In fact, these findings are not unexpected. Although the classic early work regarding EAE studies established that CD4+ T cells were the critical effectors driven the disease, soon it became clear that B cells and their antibody products have essential roles too. Willenborg and Prowse demonstrated that rats with B lymphocyte depletion by administration of anti-IgM failed to develop MBP mediated EAE [20] Moreover, these anti-IgM treated rats, resistant to EAE, develop EAE symptoms when treated with anti-MBP serum [21]. Weber and colleagues induced murine EAE by recombinant MOG (rMOG), a model in which B cells are considered to contribute pathogenically, or MOG35–55 peptide, which does not require B cells. In the model induced by rMOG, B cells became activated and as antigen presenting cells (APCs), promoted differentiation of proinflammatory MOG-specific Th1 and Th17 cells. B-cell depletion with anti CD-20 treatment prevented or reversed the EAE, which was associated with less CNS inflammation, elimination of meningeal B cells, and reduction of MOG-specific Th1 and Th17 cells. All these findings together suggested distinct roles for B cells in CNS autoimmunity and the importance of the differences in immune responses to MOG protein and peptide when choosing an EAE model for testing novel B cell-targeting agents for MS [22].

The at-EAE model presented here could be a useful tool for studying the role of B lymphocytes in the pathogenic mechanisms of the MS and for testing potential new therapies focused in B cells and humoral immunity. Other adoptive transfer models that use MBP as encephalitogenic peptide concluded that the disease was not dependent upon the presence of B cells and antibody production [21], without taking into account that the pathogenic role of antibodies in EAE studies depends on the specificity of the antibodies. MBP has an intracellular localization while MOG cell surface expression makes it accessible to antibody recognition, reason why MOG could be necessary in order to have B cells and antibody production in our at-EAE model. Other MBP EAE passive models in rats used MOG antibody injections in order to study the role of autoantibodies in the pathogenesis of MS [23], [24], but in these models the antibody is injected and there are few B cells present in the CNS making difficult the study of any direct role of B cells in the pathogenesis that is not mediated by antibodies. Is crucial taking into account that, apart from secreting demyelinating antibodies, B cells present myelin autoantigen by concentrating local autoantigen with their immunoglobulin receptors [15], [25]. B cells also contribute to create a particular cytokine environment, being able to produce interleukin 10 (IL-10) [26], [27]. IL-10 is a pleiotropic cytokine that exhibits potent anti-inflammatory activity [28]. The mechanism implicated, location and cellular targets of B cell-derived IL-10 regulation in EAE are not known because low numbers of B cells enter the CNS during disease. The high amounts of B cells found in the inflammatory infiltrates in this model make it useful to study the role of B cells in the pathogenesis of the disease.

Moreover, the model described in the paper allows the study of all the pathogenic mechanisms related with the antibody production by B cells but also other possible neurodegenerative mechanisms not antibody-related as for example T:B cell interactions. In this sense, as the model is developed under a C57BL/6 background, transgenic variants are possible in order to answer specific questions.
